# Compound Comminuted Fracture of the Humerus

**Published:** 1858-03

**Authors:** John Meckley

**Affiliations:** Reading, Pa.


ART. V.—*Case of Compound Comminuted Fracture of the Humerus: excellent recovery.* By JOHN MECKLEY, M.D., of Reading, Pa.

THE unnecessary frequency with which amputation is resorted to after severe injury to the bones and soft parts of the extremities, more especially by country practitioners, who, rather than run the risk of a deformity for which they might be prosecuted in the courts, are oftentimes led to sacrifice a limb which, by careful dressing and close watching, might be preserved, induces me to present the following case as illustrative of what may be accomplished in this way, in the absence of many of the conveniences which city surgeons have at their command.

Dec. 17, 1854, J. C. O., aged twenty, a bridge carpenter, fell from a railroad bridge upon the ice below, a distance of thirty-three feet, striking, in his descent, several times upon the timbers of the bridge. He was taken up insensible, and it was observed that the arm bone was broken just above the elbow, the upper fragment protruding through the two shirts and two coats that he had on at the time. Three or four fragments of bone were also picked up, the largest measuring nearly two inches in length, three-fourths of an inch wide, and about one-third of an inch in thickness. I reached the patient about an hour after the accident; he had not fully reacted, but was complaining a great deal of pain in his back and hips, and numbness of the injured arm. I found, on examination, that the humerus had suffered fracture just above the elbow-joint, but not involving the latter, and by pressing the parts between the thnmb and forefinger it was ascertained that comminution had occurred to the extent of about an inch and a half. The wound of the soft parts was situated half an inch above the internal condyle, and upon introducing the extremity of my little finger I could readily detect the existence of loose fragments of bone. Hemorrhage was very slight.

After the administration of half a grain of sulph. morph., dissolved in a glass of wine, I applied the angular splint, made of felt cloth, after the method recommended by Professor Hamilton, of Buffalo; Dr. Erster, at whose house the patient was staying, assisting me in the dressing, and otherwise aiding me by his excellent advice. The patient not being able to lie down, in consequence of the other injuries which he had received, was placed in a large rocking-chair, the arm resting upon pillows, and supported by a small table placed conveniently near, and cloths wet with a mixture of one part of Goulard's extract, one part of whisky, and two parts of water, kept in contact with the limb at the seat of fracture. He was obliged to remain in the chair for six or eight days, and for the first day or two enjoyed but little rest.

On the third day I found considerable swelling, and on removing the dressings a good deal of oozing of bloody serum was observed. Upon reapplying the apparatus, I left an opening opposite the seat of fracture, and directed lint steeped in tinct. arnicæ to be placed upon the wound.

On the fifth day the patient's general health had considerably improved; he had slept well for two or three nights previously, but for the last twenty-four hours had suffered a good deal from a burning pain and sense of crawling in the wound. On removing the lint from the latter a number of large maggots were seen moving actively about, although the weather was very cold. The only way in which I could account for the presence of these animals was upon the supposition that they were developed from eggs deposited in the cotton used for padding the splints. The wound was cleansed by injecting tinct. arnicæ and water, and flax tow substituted for the cotton.

From this time the case continued to do well, and on the 4th of January, eighteen days after the accident, the wound in the soft parts having healed and the union of the bone progressing rapidly, I allowed the patient to be taken in a sleigh to his home, in Luzerne County, a distance of eighty miles. Three weeks afterward he was able to write me a letter with the hand of the fractured arm, and six weeks subsequently he called at my office. I then found that consolidation was complete, and the functions of the elbow-joint entirely restored, excepting inability to extend the forearm to a perfectly straight line.

Owing to the departure of the patient at the time mentioned, I had not an opportunity of employing passive motion during the treatment.

